# Korea’s Early COVID-19 Response: Findings and Implications

**DOI:** 10.3390/ijerph18168316

**Published:** 2021-08-05

**Authors:** Shin-Kue Ryu, Soon-Gwan Chung

**Affiliations:** 1 Department of Political Science, Idaho State University, Pocatello, ID 83209, USA; ryushin@isu.edu; 2 Department of Public Administration, Sunchon National University, Suncheon 57922, Korea

**Keywords:** COVID-19, South Korea, government, pandemic, contain

## Abstract

South Korea was a hotspot of the COVID-19 pandemic with confirmed infections quickly surpassing 10,000 people. However, the country quickly responded and contained additional infections with minimal costs of lives. Hence, the question, “what did they do differently?” Building on empirical fingerprints from over 1507 pages of South Korean government press briefings on their public sector response between 31 January 2020 and 1 July 2020, we capture the sufficiency-based mechanism in operation with two key findings. First, mechanisms matter in pandemic containment, i.e., sequence, complementary activities, and systematic settings are consequential to the witnessed outcome. Second, central government-led efforts were effective and in parts necessary to deal with invisible and rapidly spreading infections beyond a single jurisdictional boundary. These findings lead to a timely discussion on whether pandemics should be treated in the same scholarly limelight as other natural disasters.

## 1. Introduction

**Figure 1:**
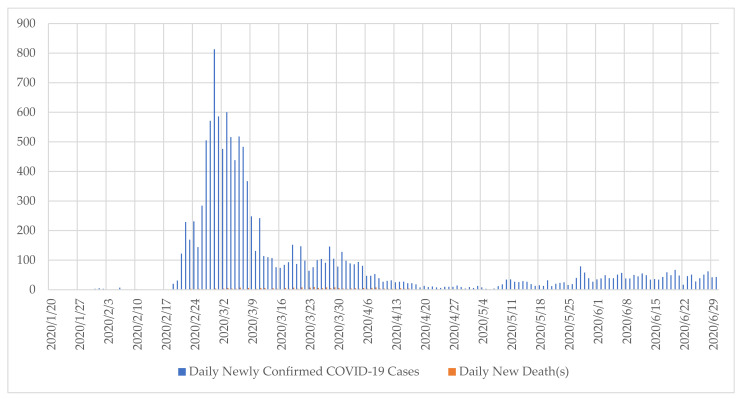
South Korea COVID-19 Infection Trends from 20 January 2020 to 30 June 2020. Source: Compiled with dataset created from the series of daily reports issued by the Ministry of Health and Welfare on its status of COVID-19 figures. The series is in the form of KCDC (2020). 코로나바이러스감염증-19 국내 발생 현황 [Coronavirus-19 Domestic Infection Rates]. Seoul: Ministry of Health and Welfare.

Due to China’s geographical proximity and its status as the largest trading partner, the South Korean Government was on high alert over the possibility that China’s reported Coronavirus Disease 2019 (COVID-19) may travel over into the country [1]. Despite being aware of the risk, South Korea was not immune to its spread into its borders. It reported its first COVID-19 confirmed case on 20 January 2020 [2] and soon after infections spiked as depicted in Figure 1. With 81% of the country’s 51.7 million population residing in the urban areas [3,4], the contagion was a matter of where, when, and what magnitude once it entered the county. By February 2020, South Korea had the second most reported COVID-19 cases in the world outside of China with cumulative infection cases in thousands [5].

South Korea’s COVID-19 infection rate patterns started to change in March and remained flattened. By containing the rise of mass infections, the country moved down the international ranking of COVID-19 infections [6]. This raises the question on South Korea’s pandemic response system and its operations, i.e., what did they do differently that warranted such a different outcome to move it towards an outlier case?

The study builds on the governments’ 135 daily official press briefings between 31 January 2020 and 1 July 2020. The presented government’s pandemic response model is the result of translating all these press briefings, writing the corresponding narrative of events, and carrying out analytical distillation to capture the response mechanism. Narratives is a trusted method for this research question since it highlights the dynamic and sequential aspects of temporality [7]. The first half of 2020 was set as its temporal boundaries since the timeline captures the spike, response, and prevention of COVID-19 infections. More importantly in April and in response to declining new COVID-19 patient numbers in the country, the South Korean government closed the 18 residential treatment centers due to declining COVID patients in the country. The residential treatment centers were opened in early March to temporarily expand treatment capacity to isolate and treat light symptom patients.

The qualitative nature of the employed empirical data combined with the study’s objective to capture longitudinal institutional responses makes it suitable to employ Beach and Pedersen’s process-tracing method as its research design [8,9]. Within their different types of processing tracing methods, this study adheres to the methodological guidelines for ‘explaining-outcome process tracing’ since the questions pertain to uncovering and identifying the mechanismic explanations behind a relatively successful Government response to the COVID-19 pandemic. The “mechanismic” term was first coined by Beach and Pedersen refers to the “understanding of causation that focuses on the process whereby causal forces are transmitted through a series of interlocking parts of a mechanism to produce an outcome” [8].

The research design entails an iterative process between deductive and inductive paths of inquiry. Surveying existing scholarship on known public health and pandemic response frameworks helps articulate the mechanism constructed via an inductive approach of utilizing ‘fingerprints’ of collected empirical data. A subsequent section elaborates on the data and method employed for this study. The final sections present the identified government response mechanism and its implications for scholarship.

## 2. Theoretical and Conceptual Framework

Multiple sub-streams of public administration scholarship apply to government responses to the COVID-19 pandemic. Emergency, disaster, and crisis management studies serve as the intellectual hub of that literature because a pandemic possesses similar attributes – an infrequent event beyond the mitigation capacity of a single local jurisdiction authority [10,11,12,13]. Recognizing COVID-19 is an invisible airborne virus that cannot be extracted from the transmitting medium in open space, response systems do not have full control over the behavior of the virus. Thus, the pandemic responses are typically limited to identification inclusive of investigation activities, communication, quarantine, and care delivery [10].

From the disaster phases or “life-cycles” scholarship, immediate responses are grouped into one stage within a multistage cycle of emergency/disaster management [13,14,15,16,17]. The cycle is comprised of mitigation, preparedness, response, and recovery phases. The cycle is relevant since it highlights the importance of activities outside the immediate response phase as the effectiveness of response activities during an emergency is intricately linked with actions pursued during the mitigation, preparedness, and recovery phases. Despite the relevance of activities during the other phases, the study focuses on specific activities of identification, quarantine, and recuperation that fall into the response phase in the lifecycle. This is because during 2020–2021 all governments were still squarely within a prolonged response phase on COVID-19. However, governments with experiencing varying results. Thus, the linear phases framework requires supplemental insights to capture and explain the outcome disparities in different contexts.

The disaster phases framework is still very relevant for this study as it highlights the importance of efforts and actions in the preceding phases within the feedback cycle. They are critical in the design, execution, and establishing of the enabling environment of response activities. In short, anticipatory preparations directly determine the response units’ effectiveness during a disaster. This is because response activities are set by policy designers working from their own world outlook built from past experiences and expertise [18,19]. Thus, the question is, “why are certain localities better prepared than others for large-scale emergencies?”. The inquiry, when applied to public health crises such as influenza, often points to individual-level determinants such as risk perception [20,21] and its complementary factors of stakeholder characteristics [22]. In the United States, it has been found that responses to public health crisis such as H1N1 pandemic relies on voluntary compliance and recommended behavior changes [23].

These studies on individual perspectives and stakeholder characteristics are especially relevant in democratic settings as the public entities involved in mitigation and preparedness are beholden to a political process built on preserving civil liberties [24]. Thus, government response strategy varies on institutional arrangements and cultural determinants [25]. The institutional arrangements are political products since budget prioritization involves a political process [26]. Furthermore, legislative behaviors in a democratic setting prioritize exigent crises [27]. Thus, an institutionalized setting before the onset of a pandemic is a product of public sentiments and desires manifesting into semi-permanent concrete policies, organizations, programs, and projects. A process that by nature involves a broader set of stakeholders [18,19] via multiple paths [28] dependent on settings. The process often involves a heavy-handed intervention and input of technical experts within the bureaucracy [29]. However, the latter is not the only voice in the room or policy-making path [28].

Although public institutions’ involvement in public health crises is fitting, the actual mechanics of the intervention is complicated, at least in the case of the United States, due to multi-actor involvement in public health delivery [30]. In the United States, an additional layer of complexity exists due to preventative health care systems having historically developed separately from responsive health care systems [31]. An evolution that is further complicated by the various paradigms grabbing hold of the U.S. federal government and its corresponding lasting imprints on publicly sponsored health programs [32,33,34]. The diversified landscape on the means of public service delivery is a product of the various tools of government available in public policy design in the U.S. setting [30,35] and its mix, particularly the use of purchase-of-service contracting involved in healthcare delivery [36]. An end-product of the past is the landscape of public service delivery that is both varied and interconnected, or loosely connected; a latter characterization resulting from implementation delegation that is often imposed yet unfunded [37].

The different historical legacies of public institutions in different settings place the onus on public health on different entities. Some place great weight on the individual as highlighted in individual-level studies [20,21,22,23]. Others place greater weight on the public sector response [10,11]. No studies disregard the importance of both the individual and government response. All emphasis is a balance of the two. The question then is why do we see variance? This study serves to advance the literature by focusing on the government response of South Korea that was relatively effective in containing the contagion after COVID-19 landed in the country. The study aims to identify the response mechanism and expand on its implications to both practitioners and scholars.

## 3. Data Set

The Ministry of Health and Welfare’s Central Disaster Management Headquarters (MHW CDMHQ) of South Korea held daily briefings on government responses to the COVID-19 pandemic starting from 31 January 2020, which was a day after the first registered domestic infection case. The first COVID-19 case was of foreign origin that was confirmed on 20 January 2020. The briefing responsibilities were later transferred over to the Ministry of the Interior and Safety’s Central Disaster and Safety Response Headquarters (MIS CDSRHQ) on 25 February 2020. The style of reporting did not change as the transfer reflects the broadened involvement of all government entities in the pandemic response. Between 31 January 2020 and 1 July 2020, 135 public announcements of a combined 1507 pages took place. All daily briefings were acquired via the Korean Government website. All briefings are translated into English by the manuscript researchers and are available for download at http://www.shinkue.com/post/data-for-covid-19-journal-article, accessed on 30 July 2021. 

**Figure 2:**
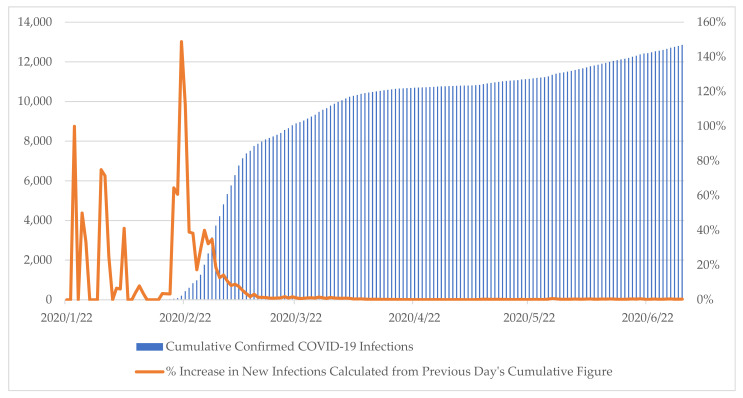
Korea COVID-19 Infection Trends from January 20, 2020, to July 1, 2020. Source: Korea Ministry of Health and Welfare. Coronavirus Disease-19. Available online: http://ncov.mohw.go.kr/en/ (accessed on 21 May 2021).

The cutoff date is set at 1 July 2020 since it reflects the date to which it becomes undeniable that the spike in infections has been flattened as shown in Figure 2. Furthermore, the government dismantled institutions specifically set up to address the spike by the data cutoff date. Government briefings continue beyond the cutoff date. However, for the purpose of the study, the analysis is restricted to the early half of 2020. The contagion was contained as demonstrated by the percentage increase in new cases. 1 July 2020’s new case number accounts for only 0.39% of the cumulative total number of cases. The corresponding 5-day average of the same figure is 0.39%. This percentage increase figure shares the underlying logic resembling the Basic Reproduction Number (R0) used in epidemiology to depict the innate infectiousness of the disease [38].

Since the study is interested in the interventions that led to suppression of infection agent’s transmission despite the disease’s innate contagiousness, the simpler figure depicted in Figure 1 is sufficient. Most notably, 20–22 February is of particular interest since that period exhibits exponential growth in contagion indicating an accelerated spread of the virus following the contagion radiating out of a Shincheongi church gathering. Thus, the dataset is appropriate in identifying the responses that nipped the accelerated growth of infections and follow-up institutionalization efforts put in place to prevent relapse to spikes in new confirmations. The briefings dates also cover the Etaewon nightclub contagion in April [39].

Because the data set is the starting point of the process-tracing research design employed in this study, the question is whether the data is a trustworthy source for evidence in the empirical evaluation. For this purpose, Beach and Pedersen suggest the following litmus test of five conditions that the source should satisfy [9]:

*(1)*  *Can the source have known about the events?*
*(2)*  *How many steps removed from the events is the source?*
*(3)*  *Is the source reliable?*
*(4)*  *Does the source have motives for distorting content?*
*(5)*  *How can we cross-check to increase our confidence in accuracy?*

The data satisfies all the conditions. First, it knows about the events since it is a government briefing summarizing the pan-government response to the pandemic. Second, the source is zero steps removed, it is self-reporting of government initiatives and activities. Third, the source is reliable as it is competent to observe what took place and provide an accurate account of it. Beach and Pedersen define “source reliability” in terms of source competency in encapsulating and communicating observations [9].

Fourth, government sources are typically approached with a healthy dose of skepticism. The skepticism stemming from the possibility of selection bias to fit the narrative of its authors [40]. Thus, the fourth test is tied with the fifth test of cross-checks for accuracy. A check that questions freedom of the press and whether a competitive democracy is at work. The availability of alternative reporting to corroborate government communication along with the ability for opposing political parties to host public debates with a possibility of redemption and reward over its claims via competitive elections improves confidence in source accuracy as multiple independent sources exist for cross-verification.

Indicators reflecting this contextual setting are available. The case study country has a relatively free and diverse press in a democratic setting where it is ranked 42nd out of 180 countries in the 2020 World Press Freedom Index [41] and is ranked 23rd in the world in EIU’s 2019 Democracy Index [42]. For reference, the United States is ranked 45th and 25th, respectively in these indexes. More importantly, the government sources in this particular case carry a higher probability of accuracy in this particular case study since transparency is a strategy for pandemic containment [43]. As a public health emergency latches onto fear and triggers fearmongering, it was crucial to have a reliable and regularly updated source of information regarding the status of the pandemic and for citizens to trust government-led initiatives.

## 4. Methodology

The ‘explaining-outcome processing-tracing case study method’ is employed [9]. It is fitting since the study is interested in capturing the mechanism at play that is triggered by a cause to achieve a specific outcome. Given mechanisms linking cause to an outcome are operative within cases rather than across cases, cases are a preferred platform and approach [44]. The cause here is the respiratory public health emergency and the outcome is containing the respiratory public health emergency. South Korea’s pandemic response from 31 January 2020 to 1 July 2020 fits the case study selection criteria and case definition since it is the unit that contains the causal relationship. The temporal boundaries are appropriate as they encapsulate the chronological developments of outbreak and containment as evidenced in Figure 1 and Table 1. The latter illustrates the milestone where a greater number of patients are discharged from quarantine than are positive COVID-19 patients being admitted to medical facilities.

Separating the mechanism from cause is necessary since “mechanisms are not causes but are causal processes that are triggered by causes and that link them with outcomes in a productive relationship” [9]. This approach is preferred over variance-based approaches due to innate inaccuracies that arise from reducing theoretically unique and critical components into numerical form. Furthermore, the strict variance-centric epistemological approach has limitations in assessing public administration performance as police and fire departments continue to reform despite flat crime and fire rates [45]. In this case, the variance-centric approach will not capture the innovations in the response mechanism that contributed to the continued suppression of new COVID-19 cases even after it was flattened. Innovations were necessary in light of evolving circumstances and moving targets. Thus, rather than approaching the study from an ontological probabilism approach where across-case variation is the emphasis and focus, the study adopts the ontological determinism approach where it seeks to identify the reason behind a materialized and verified outcome [46,47]. In this case, South Korea was able to flatten its COVID-19 spike and kept it flat while other countries were finding it challenging to do so. As the approach requires aggregating mechanistic evidence in support of inferences on causal relationships within a specific case, it is context specific. Thus, it requires the identification of requisite contextual conditions that were essential for the mechanism’s operation.

Historical conditions matter in a causal mechanism, particularly more so when the mechanism involves studying the government apparatus, as it becomes the unique embodiment of ebbs and flows power emanating from legacy structures, fluctuating societal exigencies, and deliberation [48,49,50,51,52,53,54]. Thus, the mechanism description and discussion need to be nested, yet distinguished, from the requisite contextual conditions. A necessary distinction for a more accurate generalizability discussion.

Due to the operational interlinkage between mechanism and context, mechanismic explanations take on a context-dependent characteristic and are characterized as heuristic systems [9]. That is, mechanisms cannot be reduced to counterfactual dependencies since the removal of individual parts can either destroy or change the system [9,55]. Thus, employing an intervening variable approach as a research design in place of a mechanismic approach to identify causality of a known outcome is problematic due to its inaccurate and invalid implication that mechanisms can be described as a set of independent pliable variable figures. The approach is unsuitable since the study is aiming for the completeness of the mechanism.

As a method, process tracing reveals asymmetric causation [56,57]. This is because its only claim is the careful examination of the causal effects between a cause and an outcome by outlining the mechanism for this singular case. It does not claim what would have happened in absence of the mechanism. The method strives to achieve a comprehensive case-specific explanation with high internal validity over a generalizable theorized mechanism. This is particularly salient in studying COVID-19 responses, as certain vital contextual conditions are already predetermined. The number of hospitals and the availability of trained healthcare professionals with lengthy educational requirements is not something that can be switched up and down based on convenience. The health insurance landscape that determines availability, coverage, and operations with other healthcare entities are shaped by legacies involving past legislation, regulations, and investments. This is more pronounced in the South Korean case due to its long legacy of a national health insurance scheme that started in 1977 [58]. Citizen receptivity and trust of public sector announcements on public health is emboldened or attenuated by the recollection of past interactions. All of which is path-dependent on historical state-society relations.

Of different variants of the process-tracing case study method, this study adheres to the explaining-outcome process-tracing as depicted in Figure 2. Explaining-outcome process tracing’s main focus is to craft comprehensive explanations to provide a heuristic insight into the causes behind an already manifested specific outcome. Because identification of the mechanism with sufficiency as its foremost criteria is the goal, detailing the narrative of events is a natural starting point as it highlights how the public sector institutions responded to a moving target. The narrative of events provides hints to the causal process with leads to uncover the operation of an unknown mechanism [9]. Given the abundance and richness of information, sifting the relevant evidence from all available evidence is necessary. Here “relevance” working off a U.S. legal definition and referring to evidence consequential to assessing the probability of certain actions considering the fact [59]. In accordance, a narrative of events was constructed with evidence sifted for its containment of respiratory public health emergency. That is, the evidence-based construction of the causal mechanism is conditioned by its relevance to the outcome, a condition described as “productive continuity” [60]. Given the research is interested in bringing focus to the operating causal mechanism in the case study, temporal order was important where each associated observation is time-stamped. In accordance with the explaining-outcome process-tracing research design good practices, the study constructs a sufficiency-based understanding behind the asymmetric causal mechanism responsible for the known outcome. This type of research design is effective in identifying an underlying mechanism at play as demonstrated in Tannenwald [61].

## 5. Analysis

The presented analysis is narrowed to the outcome-explanation process-tracing mechanism and is confined to the work after the construction of the evidence-based narrative of events. The compilation of sources comprised of 1507 pages, translation, and categorization of the qualitative data, and the construction of a narrative of events is available for download for verification from http://www.shinkue.com/post/data-for-covid-19-journal-article. Due to the time-consuming nature of the entailing multistage due diligence process, detailed discussions on the earlier stages of the research design are omitted from this article since they can be verified by downloading the data set. It is acknowledged that all presented analyses are consequential of those early efforts. Thus, any verification of veracity and methodological appropriateness should start there.

**Figure 3:**
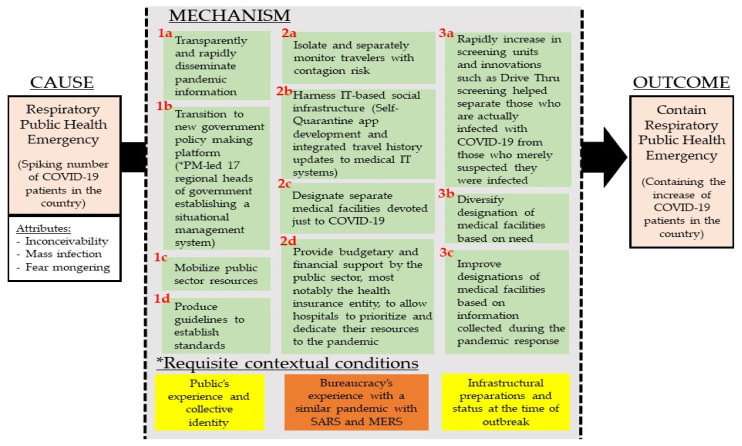
South Korea COVID-19 Response Causal Mechanism Diagram.

Figure 3 depicts the mechanism constructed from the narrative of events. The cause/trigger is the respiratory public health emergency. It carries the attributes of inconceivability, as the virus is invisible to the naked eye. It carries the potential of mass infection. It is able to invoke fear by the public. Identifying the cause with attributes is methodologically relevant as Beach and Pedersen articulate it allows “a better idea of what facets of our cause triggered the mechanism and what it eventually produced, enabling us to focus our theorization on relevant causal logic that can bind together the cause and outcome through a mechanism” [9].

Each component part in the mechanism is extracted based on either their theoretically unique or critical role in the productive continuity in relation to the outcome. Table 2 lists the empirical fingerprints of each component by aggregating mechanistic evidence from the dataset. The layout and number of the components are in reflection of a logical spread rather than a chronological spread. This is because a chronological array is already provided by the accessible narrative of events, and it is more important to identify the theoretically unique or critical features for the purpose of identifying the mechanism. The latter criteria better uphold one of the outcome-explaining process-tracing objectives, which is to understand how the outcome was attained (an explanation that is anchored on sufficiency in the productive continuity).

The exercise of arriving at a sufficient explanation was cross-examined by additional references on normative responsive actions. Of particular, Landesman’s work published by American Public Health Association [11] outlines the five unique components of the public health response to unknown disease outbreaks as follows:

*(1)*  *Detection of unusual events*
*(2)*  *Investigation and containment of potential threats*
*(3)*  *Organization of care*
*(4)*  *Laboratory capacity*
*(5)*  *Coordination and communication*

The empirically derived mechanism outlining the “how” in South Korea’s case covers the main grounds corroborating and confirming its soundness to existing U.S.-centric emergency response literature but also highlights its current insufficiency in capturing modifications and innovations resulting in a different outcome. For example, “detection of unusual events” is systematically strengthened by updating epidemiological information systems and adopting unique processes to improve detection. In Korea, an Epidemiological Investigation Support System (EISS) [62] was in operation supported by the Infectious Diseases Control and Prevention Act, which at the time of COVID-19 was the 64th amendment to the 1957 Infectious Disease Prevention Act [63] and is a product of the leadership’s on-going prioritization of public health [64]. The Act’s Article 2 Section 7 requires the government to set up plans to manage infectious disease information using ICT. This is because Article 7 Section 2 on *Measures to Stop Contagion* requires the government to disclose all information necessary for citizens to exercise preventative action. This including disclosing information on routes, transportation means, contact status, etc. to the public when it exceeds just merely warning status. To do so, the Act authorized the collection of relevant data for rapid tracing such as credit card information and cell phone data service usages. The Act was amended in March 2020 to better protect patient’s rights to enhance voluntary cooperation by the public. The government mentions the use of such data on their May 12 and 13 briefings in their attempt to contain spread originating from a nightclub. The innovation in the example is the system that was set up to enhance policy implementation effectiveness. This is integrated into component 2b of the response mechanism. The exercise of linking empirical evidence with the components is highlighted in Table 1.

The identified mechanism highlights some expected components. Investigation and containment of potential threats were enabled by strengthening testing capacity, mask distribution, and public reminders on its use. Yet, there were additional efforts. The government was actively involved in disseminating standards and establishing standards with guidelines and public announcements. Trafficking of care took place via designated medical facilities. Specialization was emphasized with complementary efforts to reinforce response capacity. Protective equipment was mobilized to prioritize frontline healthcare workers. Immediate reforms in the National Health Insurance programs took place to ensure financial sustainability and to incentivize volunteer participation. Laboratory capacity was strengthened with increasing production of the testing kits from one company to two. Thus, the increased availability of testing kits and improved turnaround times for testing itself, and dissemination of results using information technology provide positive feedback benefits to identification, containment, and treatment. Tying all this together was coordination and communication whereby protocols were established early with the transition to a joint government policy-making platform and routinized daily briefings.

**Figure 4:**
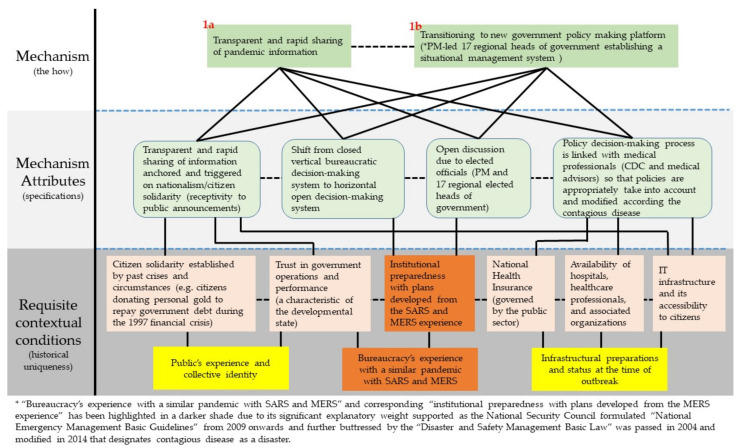
Diagram elaborating requisite contextual conditions.

Identifying requisite contextual conditions adds the “why” explanation to the “how” explanation outlined by the mechanism. The difference between the two is where the “how” identifies what pieces of the mechanism were present is part of the trigger-mechanism-outcome causal train. The “why” requires deeper investigation into the reason behind the presence of the particular mechanism in the specific context at the onset of the pandemic. Its demonstration is best illustrated by elaborating on the two early responses to the disease outbreak: (1a) flexible and rapid decision-making and (1b) formation of a new government policy-making platform as illustrated in Figure 4. Specifying the requisite contextual conditions is necessary as possession of a response mechanism blueprint does not necessarily translate to its manifestation and effective operation. In South Korea’s case study, it can be largely reduced to (1) public’s experience and collective identity, (2) bureaucracy’s experience with a similar pandemic with SARS and MERS, and (3) infrastructural preparations and status at the time of the outbreak. South Korea is a largely homogeneous society with a common historical experience, which is an experience that is geographically confined being located on a peninsula and with a common history proliferate with national solidarity eliciting events. This condition defines the citizen response and the social norm to the mechanism.

The status of infrastructural development matters as it sets the response capacity parameters. Hospitals, Community Healthcare Centers, Call Centers, and the healthcare labor force cannot be conjured overnight. IT infrastructure, inclusive of public health-related data management systems and citizens’ IT connectivity, varies by country and is relevant in receiving and verifying health announcements. South Korea, fortunately, had one of the highest interest connectivity rates with 96% of the population with access to the internet as of 2018 [65]. The IT infrastructural factor is crucial as the country makes active use of smartphone possession such as the ‘자가격리자 안전보호 [Self-Quarantine Safety Protection App]’ and ‘전자출입명부 [KI-Pass].’ The latter app uses anonymous QR code generation on individual cell phones to automatically populate electronic entry lists to improve the accuracy and tracing response time of epidemiological studies. Both are downloadable at the Apple App Store and Google Play Store.

South Korea’s relatively effective control over the spread of COVID-19 confirms preparedness does indeed enable responsiveness. The experience of MARS instigated recovery and mitigation efforts to prepare for similar pandemics. This raises the concern on the importance of separating requisite contextual conditions from the mechanism. An analytical process that is vital to other countries dealing with a similar crisis as it asks whether the mechanism is relevant and feasible in different settings such as the United States where “local primacy” is a key principle [13].

Actionable triggered response measures of South Korea include establishing a coordination platform and designating a health professional public entity to follow-up on mobilization efforts. CDMHQ led by the Minister of Health assumed this role in Korea and was later superseded by the Prime Minister-led CDSRHQ. This established early on a unified voice on public sector response to the respiratory pandemic with its transparent and rapid dissemination of pandemic information. With the launch of online sites, enabled accessibility to daily briefings, and clarifications on misleading or misconstrued information, the public sector was able to dictate a unified healthcare narrative. This was important since the country has over 50 million people exercising free speech and free press. Personal interpretations and non-scientific conspiracy speculations could hijack and undermine recommended voluntary compliance actions disseminated via public announcements. Due to the invisibility of the virus without scientific tools, it was vital to share instrument-aided detection results of COVID-19 infections with the public and to have the public trust the message that the healthcare professionals are sharing with the public. Correspondingly, budgets were mobilized for local governments to assist in their public announcement delivery.

Establishing the science-based narrative on COVID-19 early on carried an additional benefit of shaping people’s voluntary activities and actions that helped curb the spread of the disease. The Shincheongi church outbreak and the Etaewon nightclub outbreak illustrate this aspect. Scientific explanations based on virology and epidemiology were more convincing explanations than rival explanations based on alternative world views. Risk perception of the virus was definitively set by the healthcare professional-led public sector. Hence, it effectively quelled any doubts over the threat level of the virus. The perception was complemented by action, as the government-led efforts in supplying and stabilizing masks to industries and entities early on, further encouraging abidance to the public sector recommendations.

**Figure 5:**
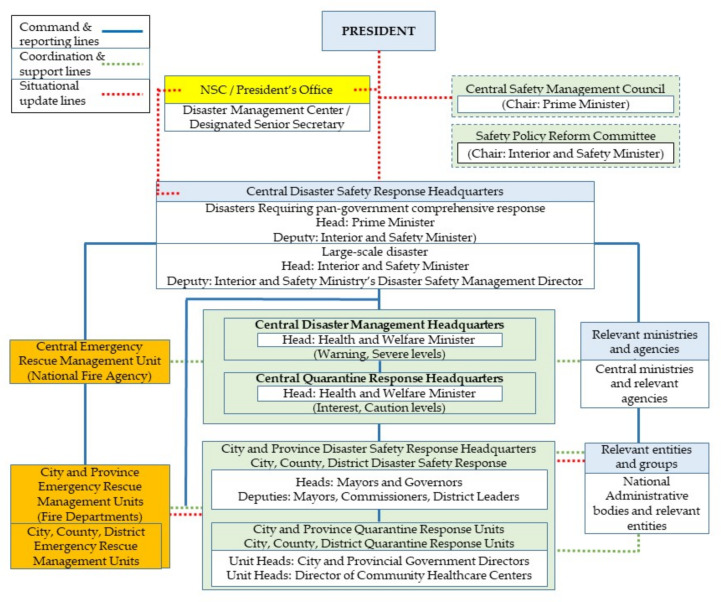
Disaster Management Reporting, Coordination, and Situational Updates Arrangements.

Institutionally, South Korea provides valuable lessons. First, it quickly transitioned to a central government-enabled emergency government policymaking platform as depicted in Figure 5. This includes establishing clear reports, command, coordination, and communication lines. More importantly, it is characterized by a shift from a closed vertical to an open horizontal bureaucratic decision built on open yet scientifically anchored discussions with policy formulation and implementation entities and healthcare professionals. The platform allowed a rapid ramping up of government response, both in terms of evidence-based decision-making to abating citizen concerns via scaling up call center operating capacity. The offline decision-making platform established early their mode of operation by sharing pandemic information online as transparently and rapidly as possible.

The central government quickly assumed the core nodal role of compiling relevant information and established operational standards via issued guidance from updated information. In regard to quarantine, recommended practice compliance was monitored in cooperation with local governments. Expectations of acceptable standards to be inspected were shared with the relevant entities. Sharing this information publicly also served as a reference point to report any violations. Thus, it served as a baseline in the arbitration to resolve any disputes among citizens and monitoring entities. The new decision-making platform built in a process for constant updates to the guidelines via stakeholder consultative process. This allowed the timely resolution to the concerns raised during the implementation of government guidelines.

Subsequently, the government engaged in series of activities categorized as the 3T’s: Testing, Tracing, and Treatment [66]. On tracing, they rapidly increased screening units with innovations such as the Drive-Thru triage to help separate those who are infected with COVID-19 from those who merely suspected they were infected. The rapid and accessible testing aided in dispelling fear from the unknown and preventing uncalled-for panic in healthcare facilities. This was enabled by rapidly ramping up the production of testing kits with more kit manufacturing companies in the supply chain.

Testing was complemented by tracing. This involved isolating and monitoring travelers exposed to infection risk. A special entry process at the ports and airports was designed. It included symptom checks on entering passengers followed by a mandatory 14-day self-quarantine requirement. To ensure self-quarantine compliance, dedicated monitoring staff were assigned in coordination with the local government of the entering passengers’ residence. Mobile phone applications with self-reporting, tracing, and nudging features were developed to make voluntary compliance convenient [67]. Transportation support was put in place designed to preserve quarantine during the passengers’ move to their final domestic destination to exercise self-quarantine for 14-days.

Treatment was specialized to optimize. This includes categorizing medical facilities based on COVID-19’s evolving circumstances. They designated hospitals dedicated to contiguous diseases to lead the treatment of COVID-19 patients. By trafficking COVID-19 patients to designated specialized hospitals, it minimizes the exposure of uninfected hosts to infected hosts. This includes establishing procedures and installing structures to minimize infection risk to healthcare professionals and prevent response capacity loss. These hospitals were equipped with negative pressure rooms with the public sector providing coordination support to increase its number to match the anticipated need.

Diversification of medical facility designation was necessary to ensure the healthcare system continues to function without fail. Citizen Assurance Hospitals were designated so COVID-19 negative patients in need of medical attention may still receive treatment with minimal risk of infection. This complements another innovation where designations are improved with the collected information. South Korea found that around 80% of COVID-19 patients were light symptom patients. Thus, not all COVID-19 patients required the same amount of resources for treatment. In response, Residential Treatment Centers were created. These operated in training centers and institutes with bedding capacity to house light symptom patients for quarantine. This lifted the response burden on hospitals since staffing requirements vary by severity and by redirecting light symptom patients, it frees up essential hospital medical staff to focus on severe and critical cases.

Hospitals, which are public health response organizations, operate with market principles. This means that management requires timely orchestration of revenues and costs for uninterrupted operation. Government budgetary support enabled this by covering screening unit costs and launching financial stabilization programs for hospitals. Budgetary and financial support both in form of monetary resources and procedural modifications, most noticeably visible via the health insurance entity, ensured financial sustainability of medical facilities. Complementary, economic incentives and rewards were set to elicit volunteers to join in the all-hands-on-deck national operation to curb COVID-19 contagion.

## 6. Discussion

South Korea’s early response to COVID-19 updates both best practices and stirs conventions in theory. First, at the broad conceptual level discussion on practice, its response revolving around 3T’s (testing, tracing, and treatment) does not deviate from prior guidelines [11]. However, the study finds the specific technical executions and complementary enabling innovations for effective implementation carry greater explanatory power than normative guidelines. The productive continuity of a causal mechanism only comes into focus with careful evidence-based examination on the precise execution of those normative guidelines.

The study delves into the in-depth discussion on pivotal components by employing the explaining-outcome process-tracing case study method. Sequence matters, complementary actions matter, and requisite contextual condition matters. For example, piecemeal components are evident in the U.S. [68] but individually they do not generate the same results seen in South Korea. The systematic mechanistic approach working up from rich empirical evidence presented here furthers the discussion on the precise success factors behind the country’s success in its response to COVID-19 [69]. Findings here respond to the field’s call to generate an in-depth understanding of varying performances [70].

For emergency response practitioners, the study and its evidence-based findings expand the discussion of whether the specific actions and their associated innovations should be included in the response repertoire. This includes answering meaningful questions such as whether IT-based tracing methods be adopted, whether specialization of healthcare systems during pandemic response should be adopted, whether the government should be actively involved to prevent the cornering of the market for masks, etc. There are concrete practice innovations that warrant discussion in improving the existing pandemic response mechanisms of different countries. The study also adds to the sensitive discussion of jurisdictions, authorizations, and legally bound procedures; a discussion that requires anchoring on context, as South Korea’s response cannot be discussed in isolation of its stronger central government led coordination [71] rather than locally led coordination as seen in the U.S. [72].

As the discussion expands into systems, a conceptual and methodological discussion is inevitable. A discussion expanding on how respiratory public health emergencies differ from other natural disaster emergencies. The unique attributes of invisibility and prolonged state of emergency are unique. The additional attribute of the emergency not confined to a singular geographical point asks whether existing emergency response entities, processes, and human resources prepped for geographically confined natural disasters are suited for the task. This is also relevant since the respiratory public health emergency has rippling tremors that are felt beyond the infected patients and the healthcare system. Uninfected people working outside the healthcare industry are not immune to the pandemic’s economic and social consequences. Furthermore, a pandemic differs from a natural disaster, as the response to recovery transition is not swift. Pandemics’ response tenure is longer than natural disasters such as an earthquake or a hurricane [73,74].

The theoretical discussion reverts to the methodology employed for this study. To identify the responsible mechanism, evidence is sorted in accordance with its relevance to the triggered cause. In this case, the cause exhibits unique attributes, unlike other national emergencies. Thus, it opens the discussion on whether a pandemic response should be framed in an emergency response dominated and tailored to natural disasters. It is a distinction that the South Korean government has taken notice of and adopted accordingly in recognition of the difference between natural disasters and manmade disasters [75]. An especially relevant discussion considering few countries are responding successfully to contain the spread of the COVID-19 in their preset institutional settings.

## 7. Conclusions

This study addresses the research question of, “what did South Korea do differently to contain COVID-19 more effectively than other peer countries?” It constructs a reliable explanation based on evidence. A demand for a more thorough explanation given the rising attention to South Korea’s response to COVID-19 by multiple mainstream sources in the United States such as Washington Post and New York Times [76,77]; a demand warranted by challenges other countries face in struggling to contain the pandemic. The explaining-outcome process-tracing case study coalesces the dispersed and anecdotal thoughts on the productive features of South Korea’s COVID-19 response mechanism.

The first key finding is that mechanisms matter. That is that sequences, complementary actions, and systematic settings matter. The pivotal complementary components illustrated in the mechanism based on sufficiency and productive continuity are:

(1) Transparently and rapidly disseminate pandemic information
(2) Transition to a new government policymaking platform for quick and decisive decisions
(3) Mobilize public sector resources (e.g., masks, quarantine gear, treatment equipment, etc.)
(4) Produce guidelines to establish standards
(5) Isolate and separately monitor travelers with contagion risk
(6) Harness IT-based social infrastructure for rapid tracing and communication
(7) rapidly increase in screening units and innovations such as Drive Thru triage units to helped separate those who are infected with COVID-19 from those who merely suspected they were infected
(8) designate separate medical facilities devoted to COVID-19
(9) provide budgetary and financial support, most notably via the health insurance entities, to allow hospitals to prioritize and dedicate their resources to the pandemic
(10) designate COVID-19-free medical facilities so uninfected patients can still receive medical treatment for other chronic health issues such as cardiovascular disease and cancer
(11) improve medical facilities distribution of COVID-19 patients with newly setup quarantine centers based on updated information on the virus

Identifying the mechanism ties in with the second main finding that central government-led pandemic responses work, which is contrary to the local primacy rule in federalist countries. Due to the unique attributes of the respiratory public health emergency, the effective mechanism requires a response of expansive reach. Coordinated response initiatives require a macro-level approach beyond local governments, as infections do not recognize local jurisdictional boundaries. This finding is significant and relevant as South Korea’s mechanism operated in a particular context; context reflecting the preset institutional parameters of emergency responses capacity and locally bound processes. A finding with implications to centrifugal response mechanism countries such as the U.S. It raises the question of whether the federal government should assume a greater leading role during a pandemic rather than a supporting role.

The two main findings unveil the significant implication for the field, which is whether pandemic responses should continue to be treated in the same scholarly vein as other natural disasters, as evidenced by emergency management textbooks [13]. The invisibility, referring to the general public’s inability to detect the spread without the aid of an instrument, and the prolonged response time shares little commonality with other natural disasters that are characterized as visible, short, and geographically confined. Thus, it raises the question of the transferability of response capacity of institutional responses and arrangements that are built with natural disasters in mind. The study opens this necessary discussion by supplying a blueprint to start those discussions in different country contexts.

The study provides an updated reference point on a public sector response mechanism that works. The identified components drawn from the continuous series of government briefings serve also as key recommendations for practitioners. It should serve as a reference to practitioners struggling in their fight to contain COVID-19 within their own countries. By juxtaposing the identified mechanism to their pandemic response mechanism in their country, practitioners should find it much easier to identify what areas need improvement within their response mechanisms to contain the pandemic. However, there is a caveat since not all improvements could be formulated overnight as also highlighted in our findings when detailing the mechanism. This leads to the limitations and future research agendas.

As for limitations and future homework of this study, there are a few. First, due to the nature of the empirical dataset, this study does not make any overreaching conclusions on the replicability of South Korea’s results in a different country context. This is because that answer needs to be sought following the clear separation of requisite contextual conditions unique to South Korea that are preset by the country’s unique history and the population’s historical memory. This includes the institutional setting of a strong central government vis-à-vis local governments [71]. Consequentially resulting in the central government leading the disaster management effort and the local government accepts this role, which also rests upon its unique history of SARS and MERS response efforts and the shortcomings noticed with that experience.

Another unique contextual condition in South Korea’s unique economic capacity, which is itself a historical product [78]. The relatively high economic productivity of having a GDP being 12th in the world [65] translates to its ability to finance public sector debt in the capital markets. Then there is the population’s recent collective memory of experiencing SARS and MARS, two respiratory pandemics. Thus, with vivid retrospective memory, the public needed less convincing on the potential threats and consequences of a new respiratory disease. These initial conditions need to be accounted for in the discussions of replicability as it ties with behavioral change interventions suggested by the government. However, this does not mean that the identified mechanism should not be employed as a reference point for other countries in their attempts to improve their pandemic response policies. It just highlights that some components cannot be conjured overnight. This brings us back to thinking about the planning efforts that are needed during the other phases of a disaster/emergency management cycle.

Second, this study is restricted to identifying the mechanism triggered and operated during South Korea’s early-stage response to COVID-19. South Korea is not immune from new COVID-19 infections after 1 July 2020. However, by 15 November 2020, South Korea registered 28,546 total confirmed infections (55 per 100,000) and 493 deaths (<1 per 100,000) [79]. This starkly contrasts with the U.S. figure of 11,108,686 total confirmed infections (3348 per 100,000) and 246,013 deaths (74 per 100,000). The mechanism may not be perfect in completely stomping out COVID-19. However, it is a relatively more effective mechanism than other countries faced with the same public health challenge by the virus. In addition, South Korea’s mechanism differs from the China model that went for a complete nonporous lock-down [69]. Businesses were open, people moved around, and freedom of the press was preserved. South Korea preferred an agile response that utilized modern technology and a targeted support system for the healthcare system steered from a pandemic tailored open decision-making platform. Thus, for countries that wish to avoid a drastic non-porous shutdown approach in their handling of the pandemic, South Korea’s case study serves as an alternative.

Lastly, additional studies on COVID-19 responses in other countries will help in carrying the discussions forward. More studies will help separate flexible demand-driven and crisis responding actions from the inflexible constraints facing the country. This further clarifies what actions the countries can potentially undertake by themselves versus what it may need assistance from the international community. This study starts that discussion by identifying South Korea’s working pandemic response mechanism built on central government-led mobilization and coordination efforts. On scholarship, the study contributes to the field as it offers findings on a working pandemic response that preserves civil liberties yet is very different from the traditional federalist approach to disaster/emergency management. An alternative working mechanism is provided which then informs the community on whether a paradigm shift is needed for countries struggling under the weight of ineffective pandemic response. As Kuhn emphasizes, a good time for a paradigm shift is when observations no longer fit the theory [80]. Similarly, when a pandemic response is not working, it is a ripe time to engage in the discussion of what could have been done differently. This study helps start that discussion.

## Figures and Tables

**Table 1:** COVID-19 Domestic Cases from March 4 to March 13 (in # of people).

		**3/4**	**3/5**	**3/6**	**3/7**	**3/8**	**3/9**	**3/10**	**3/11**	**3/12**	**3/13**
Confirmed patients	Vs day before	+516	+438	+518	+483	+367	+248	+131	+242	+114	+110
	Aggregate	5328	5766	6284	6767	7134	7382	7513	7755	7869	7979
discharged from quarantine	Vs day before	+7	+47	+20	+10	+12	+36	+81	+41	+45	+177
	Aggregate	41	88	108	118	130	166	247	288	333	510
In quarantine	Vs day before	+505	+388	+491	+471	+349	+211	+47	+195	+63	−68
	Aggregate	5255	5643	6134	6605	6954	7165	7212	7407	7470	7402

Note: Shading is provided to highlight the inflection point of having more patients leaving quarantine than entering it. Source: Republic of Korea Ministry of Health and Welfare. (2020). 13 March 2020 Press Release Reference Document. Sejong, Republic of Korea: Republic of Korea Ministry of Health and Welfare.

**Table 2:** Mechanism’s Empirical Fingerprints.

Parts	Empirical Fingerprints/Evidence
1a	− Daily government briefings with its recording posted online (ncov.mohw.go.kr, accessed on 30 July 2021) ○ The daily briefing translations (31 January 2020–1 July 2020) in the supplemental documents are the evidence. − Daily engagement with the media regarding government activities ○ The daily briefing translations (31 January 2020–1 July 2020) in the supplemental documents are the evidence.
1b	− Health Minister-led Central Disaster Management Headquarters (CDHMQ) initially assumes chair in the coordination efforts in the pan-government response to the pandemic. With “serious” status in late February 2020, the Prime Minister assumes the head of the Central Disaster and Safety Response Headquarters (CDSRHQ) that supersedes CDHMQ and holds daily meetings with 17 city and provincial government heads along and 15 central government ministries. ○ 24 February government briefing is the evidence that the switch in pandemic decision-making authority arrangement took place. The pandemic countermeasure responsibility was elevated to a higher government official with broader powers. The Prime Minister chairs the CDSRHQ meetings and the new arrangement brings the Minister of Interior and Security as the Second Deputy Director under the arrangement to provide support for coordination across central and local governments. (The Minister of Health assumes the position of First Deputy Director within CDSRHQ and focuses on quarantine measures.) The formal shift in the institutional arrangement brings all government entities reporting to the Prime Minister into the pandemic containment mechanism.
1c	− Early on government funds are mobilized to secure mobile X-ray units, air tents, mobile negative pressure room air machines, cover public announcement costs, purchase of masks and sanitizers for daycare centers and schools, preparatory funds for local governments to establish quarantine response systems, to strengthen testing and examination capacity, and to incentivize paid leave for quarantine. These early resource mobilizations by the government include supplying masks to service industries, small-and-medium size enterprises employing foreigners, and small-scale construction sites. Mask distribution led by the public sector reaches all sectors and populations, with particular attention paid to vulnerable groups. ○ Evidence of resource mobilization is in parenthesis: mask distribution (31 January, 19 February, 28 February, 2 March, 16 March, 17 March, 26 March, 5 April, 19 April, 15 May), mobile X-ray equipment (1 February, 2 May), air tents and mobile negative pressure rooms (14 February, 28 February, 16 March, 2 May), public announcement costs (18 February), purchase of masks and sanitizers for daycare centers and schools (18 February), strengthen testing and examination capacity (7 February, 18 February, 25 February, 24 March, 2 May, 9 May, 10 May, 11 May, 12 May, 16 May, 22 June), and incentivizing paid lead for quarantine (17 February, 26 February). Supplying quarantine supplies to private sector-operated tourism enterprises are discussed on 28 April ahead of holidays and mass migratory movements within the country. The emphasis on mobilizing resources for vulnerable groups such as special religions, illegal residents, etc., appears on 28 April, 1 May, and 12 May. De-escalation plans were prepared as well due to continuous low COVID-19 figures following relative containment. They are described in detail within 3 May, 4 May, 6 May, 7 May, 8 May, 9 May, 27 May briefings.
1d	− The government provides a series of plans and guidelines in response to the pandemic. They include (1) COVID-19 Response and Measures Plan, (2) COVID-19 Prevention Disinfection Guidelines for Public Facilities and Large [Group] Use Facilities, (3) Guidelines for Universities on Systematic Management of Responses to COVID-19, (4) COVID-19 Mass Attendance Events Quarantine Guidelines, (5) COVID-19 Central and Local Government Events Operation Guidelines, (6) COVID-19 Contaminated Mass Facility/Multipurpose Use Facility Disinfection Guidelines, and (7) Social Welfare Facility Response Guidelines. The guidelines are updated during the pandemic. ○ Evidence of plans and guidelines are in parenthesis: COVID-19 Response and Measures Plan (9 February, updated on 20 February specifically for regional governments), COVID-19 Prevention Disinfection Guidelines for Public Facilities and Large [Group] Use Facilities (8 February, updated on 26 February), Guidelines for Universities on Systematic Management of Responses to COVID-19 (19 February), COVID-19 Mass Attendance Events Quarantine Guidelines (4 February, 12 February), COVID-19 Central and Local Government Events Operation Guidelines (updated on 26 February), COVID-19 Contaminated Mass Facility/Multipurpose Use Facility Disinfection Guidelines (updated on 26 February), and Social Welfare Facility Response Guidelines (18 February, 7 March). Compliance abidance was continuously checked by relevant responsible entities and with monitoring system innovations as found on 23 March, 24 March, 5 April, 7 April, 13 April, 17 April, 26 April, 27 April, 28 April, 29 April, 30 April, 10 May, 12 May, 15 May, 16 May, 31 May, 7 June, 8 June, 9 June, 14 June, 15 June, 22 June, 24 June briefings. Preparation and guidance for other major events such as elections (12 April), reopening of schools (27 March, 3 April, 7 April, 17 April, 24 April, 15 June), and national exams (20 April, 15 May) are undertaken by the government as well.
2a	− A special entry process is established early for entering passengers from high-infection risk areas in airports and ports. This later is expanded to all passengers entering the country and is then coupled with self-quarantine enabling facilities. ○ Evidence are 31 January, 3 February, 4 February, 5 February, 6 February, 25 February, 12 March, 17 March, 19 March, 21 March, 25 March, 26 March, 27 March, 31 March, 1 April, 11 April, 12 April, 17 April, 18 April, 27 April, 28 April, and 29 April briefings.
2b	− In conjunction with 2a, a self-monitoring quarantine app is developed and required to be downloaded by entering passengers. ○ Evidence is 25 February, 17 March, 1 April, 5 April, 11 April, 24 April, 28 April, 16 May briefings. Similarly, the ‘electronic entry list’ app is developed to boost the speed of epidemiological tracing on domestic outbreaks (24 May, 2 June, 7 June, 10 June, 24June, 1 July briefings). − Severe Respiratory Infectious Disease Monitoring System (SRIDMS) and Influenza Laboratory Sample Monitoring System (ILSAM) are updated with COVID-19 testing information. ○ Evidence is 17 February, 1 May, and 12 May briefings.
2c	− Designated Dedicated Hospitals for Contagious Diseases were setup to concentrate hospital response capacity for COVID-19 ○ Evidence is 22 February, 11 March, 23 April, 25 April, 5 May, and 13 May briefings. Adjustments to regulations and procedures for the staffing of these facilities can be found on 25 February, 3 March, 6 March, 10 March, 1 April, and 30 April briefings. There were 67 dedicated hospitals as reported within the 15 March briefing. Further budgetary support is described during 16 March briefing.
2d	− Various procedural and requirement reforms were enacted with the National Health Insurance schemes to stabilize hospital finances and to encourage volunteering by healthcare professionals with monetary compensation ○ Evidence are 19 February, 22 February, 24 February, 25 February, 27 February, 3 March, 5 March, 15 March, 16 March, 9 April, 5 May, and 13 May briefings. 26 March briefing describes fee reimbursement for quarantine managers are nursing hospitals.
3a	− A Drive Thru triage unit was set up to improve testing capacity ○ Evidence is 28 February, 3 March, and 2 May briefings. A Standard Operating Procedure (SOP) for the Drive-Thru screening units was established and discussed during 3 March briefing.
3b	− Citizen Assurance Hospitals were designated to assure citizens with immediate needs such as heart and cancer patients to use those medical facilities free from COVID-19 ○ Evidence is 25 February and 28 April briefings. Details on the specific institutional arrangement and policies are found in the translated briefing. A total of 127 CAH are designated throughout the country as reported during 27 February briefing.
3c	− Recognizing 80%+ of COVID-19 patients were light symptom patients, Residential Treatment Centers were developed to be used by light symptom patients to alleviate and optimize healthcare resources. ○ Evidence are 1 March, 3 March, 5 March, 6 March, 7 March, 12 March, 16 March, 25 March, 1 April, 20 April, 25 April, 29 April, 9 May, and 14 May briefings.

## Data Availability

Supporting data is available at http://www.shinkue.com/post/data-for-covid-19-journal-article, accessed on 30 July 2021.

## Supplementary Materials

All government briefings in the original language along with their corresponding English translation by the authors with the Narrative of Events are available online at http://www.shinkue.com/post/data-for-covid-19-journal-article, accessed on 30 July 2021.
